# Brainwave Phase Stability: Predictive Modeling of Irrational Decision

**DOI:** 10.3389/fpsyg.2022.617051

**Published:** 2022-06-30

**Authors:** Zu-Hua Shan

**Affiliations:** Business School, Central South University, Changsha, China

**Keywords:** irrational decision, quantum cognition, brainwave, interference, phase stability, predictive modeling

## Abstract

A predictive model applicable in both neurophysiological and decision-making studies is proposed, bridging the gap between psychological/behavioral and neurophysiological studies. Supposing the electromagnetic waves (brainwaves) are carriers of decision-making, and electromagnetic waves with the same frequency, individual amplitude and constant phase triggered by conditions interfere with each other and the resultant intensity determines the probability of the decision. Accordingly, brainwave-interference decision-making model is built mathematically and empirically test with neurophysiological and behavioral data. Event-related potential data confirmed the stability of the phase differences in a given decision context. Behavioral data analysis shows that phase stability exists across categorization-decision, two-stage gambling, and prisoner’s dilemma decisions. Irrational decisions occurring in those experiments are actually rational as their phases could be quantitatively derived from the phases of the riskiest and safest choices. Model fitting result reveals that the root-mean-square deviations between the fitted and actual phases of irrational decisions are less than 10°, and the mean absolute percentage errors of the fitted probabilities are less than 0.06. The proposed model is similar in mathematical form compared with the quantum modeling approach, but endowed with physiological/psychological connection and predictive ability, and promising in the integration of neurophysiological and behavioral research to explore the origin of the decision.

## Introduction

Irrational behaviors, such as the disjunction effect (Tversky and Shafir, 1992), question order effect (Wang et al., 2014b), and categorization-decision effect (Wang and Busemeyer, 2016), challenge the sure-thing principle, the law of total probability, and classical decision-making theory. Researchers have offered various explanations to address these paradoxical phenomena with two strategies: one adjusts the classical decision-making model; the other builds new paradigms.

Quantum decision theory is one of the most promising new paradigms (Bruza et al., 2015; Broekaert et al., 2020), applying the concepts and mathematical techniques of quantum theory in physics to decision making. The quantum prospect decision theory (Yukalov and Sornette, 2011), quantum-like Bayesian network (Moreira and Wichert, 2016, 2017, 2018; Wichert et al., 2020), and belief entropy quantum-like Bayesian network (Huang et al., 2019) are some of the developments that enable the quantum theory to better fit and explain irrational behaviors (Xin et al., 2019).

Meanwhile, many approaches have been proposed to overcome the limitations of classical models in accommodating the paradoxical findings. The main idea is adding extra variables to classical models to simulate these decision scenarios under high levels of uncertainty. Prospect theory, which replace expected utility with subjective expected utility (Kahneman and Tversky, 1979), is among the most renowned solutions. Other effort includes various Markov models, Bayesian network, equate-to-differentiate, and Dempster–Shafer theory, or their combination (Klir and Ramer, 1990; Li, 2004; He and Jiang, 2018b).

The disputes between quantum and classical models continue to deepen the knowledge about decision making, and some studies are trying to reconcile the conflicts and unify these two models. Moreira and Wichert (2018) concluded that the classical model with latent variables could explain paradoxical findings in the prisoner’s dilemma game. More recently, Rashkovskiy and Khrennikov (2020) showed that, in special cases, the classical probabilistic model can give the same results as the quantum model.

However, both the modified classical model and quantum theory are unsatisfactory. The latter is widely accepted to be more explanatory than the former of most irrational behaviors, but both suffer the problem of the exponentially increasing complexity. Quantum theory is mostly limited to conceptual explanations of corresponding phenomena; it lacks predictive ability and is deficient in physiological and biological relevancy (Busemeyer et al., 2015; Moreira and Wichert, 2018; Surov et al., 2019).

This article presents a hypothesis for brainwave interference during decision making and proposes a concise brainwave-interference decision model (BIDM). Preliminary analysis of existing event-related potential (ERP) data supports the psychological inherence of the model. Tested with empirical data, the proposed model efficiently explains and fits the disjunction effect of two-stage gambling and prisoner’s dilemma game, as well as the categorization-decision effect. Moreover, the stability of the interference-phase differences of brainwaves under “unknown” conditions across subject backgrounds, which Surov et al. (2019) recently found in two-stage gambling, is confirmed and extended to other kinds of experiments. The proposed model reveals that the interference-phase differences under “unknown” conditions is evolved from corresponding interference-phase differences, which is nearly constant across subject groups as well, under “known” conditions.

## Challenge Faced by Quantum Decision Theory

Quantum decision theory holds that the subject is in the superposition state of all decision options before making a decision. The subject’s cognitive state Փ is represented as vector |Ψ〉 in a complex vector space. The corresponding cognitive state of a particular decision *D is* |*D*〉, and the probability of that decision is given by squared modulus of the complex-valued overlap 〈Ψ|*D*〉, defined as the amplitude of transition between the cognitive states. If decision conditions *C_i_* are represented by vectors |*C_i_*〉 in the same vector space as decision |*D*〉 and cognitive state |Ψ〉, the amplitude of reaching decision *A* from cognitive state Ψ can be expressed as


(1) $$\langle \psi |D\rangle =\underset{i}{\sum }\langle \psi |C_{i}\rangle \langle C_{i}|D\rangle$$

The statistical probability of making decision *A* is


(2) $$p\left(D\right)=\underset{i}{\sum }p\left(D|C_{i}\right)p\left(C_{i}\right)+2\sqrt{\underset{i}{\prod }p\left(D|C_{i}\right)p\left(C_{i}\right)}\cdot \cos\theta$$

where *θ* is the interference phase. The first summand in formula (2) reproduces the classical law of total probability, while the second summand is the interference term between the two transition amplitudes.

The existence of an interference term enables the quantum model to explain irrational behaviors, but its inability to define the interference phase θ before experimentation deprived the model of predictive power. However, Surov et al. (2019) then found that the interference phase *θ* is not a free-fitting parameter but a constant (*θ* = 107 ± 7°) in all two-stage gambling experiments including those in the literature and their own test experiment. This was a breakthrough in endowing the quantum model with predictive capability. Neurophysiological substrate of quantum logic was explored constructively (Barros and Suppes, 2009; Barros, 2012; Suppes et al., 2012), but the essential relationship between quantum theory and the working mechanisms of the human brain remains unknown.

## Hypothesis

The electromagnetic theory of consciousness claims that the electromagnetic field generated by the brain is the actual carrier of conscious experience (Pockett, 2012; McFadden, 2020). Different minds have distinct, characteristic wave forms with recognizable frequencies and intensities in the electromagnetic field of the brain. Technologies such as electroencephalography (EEG), magnetoencephalography (MEG), and functional magnetic resonance imaging (fMRI) are deployed to understand dynamic cognitive processes (da Silva, 2013; Bode et al., 2019; Kraemer et al., 2020). Jain et al. (2015) explored a novel technique for 3D full-wave electromagnetic simulation of brainwaves.

Previous research suggests the existence of a correlation between the probability of a decision and the level of electrophysiological signals. For example, working memory reflects reward-based decision making (Curtis and Lee, 2010) and the values of the short electrical pulses of neuronal signals reveal the decision choices (Torres et al., 2013; Webb et al., 2020). Furthermore, ERP amplitudes are sensitive to the valence of outcomes (e.g., win/loss, good/bad) and correlated with the magnitudes of feedback rewards (Yeung and Sanfey, 2004; Hajcak et al., 2006). In addition, Van’t Wout et al. (2006) showed that the skin conductance response to unfair offers in the Ultimatum game predicted rejection of unfair offers. Hewig et al. (2011) even found that emotional valence ratings, skin conductance responses, and ERP amplitudes together explained 84% of the variance in number of rejections.

Given the above theory and evidences, this article deduces that electromagnetic waves are the carriers of decision-making and proposes the following conjectures:

- Conditions trigger corresponding electromagnetic waves (“brainwaves” in this study) with individual amplitude and constant phase in the brain;
- For simultaneously comprehensive and steady detection and evaluation by a certain unit in the brain, those brainwaves should have the same frequency, and interference should occur at the site of evaluation;
- The intensity of the resultant brainwave determines the final decision.

Let conditions *C_i_* affect decision *D* and initiate brainwaves with amplitude *A_i_*. According to the wave interference rules, the amplitude of the resultant wave can be obtained by


(3) $$A^{2}=\overset{N}{\underset{i=1}{\sum }}A_{i}^{2}+\overset{N}{\underset{j>i}{\prod }}\overset{N}{\underset{i=1}{\prod }}2A_{j}A_{i}\cos\left(\theta_{j}-\theta_{i}\right)$$

where *θ* is the wave phase.

As wave intensity (*I*) is proportional to the square of the amplitude (*I∝A^2^*), equation (3) can be rewritten as


(4) $$I=\overset{N}{\underset{i=1}{\sum }}I_{i}+2\cdot \sqrt{\overset{N}{\underset{j>i}{\prod }}\overset{N}{\underset{i=1}{\prod }}I_{j}I_{i}}\cos\left(\theta_{j}-\theta_{i}\right)$$

According to electromagnetic theory, at any location the wave intensity is proportional to the probability of detecting a photon at that location *[p(D)∝I*, *p(C_i_)∝I_i_].* Therefore, equation (4) can be transformed to:


(5) $$p\left(D\right)=\overset{N}{\underset{i=1}{\sum }}p\left(C_{i}\right)+2\cdot \sqrt{\overset{N}{\underset{j>i}{\prod }}\overset{N}{\underset{i=1}{\prod }}p\left(C_{j}\right)p\left(C_{i}\right)}\cdot \cos\left(\theta_{j}-\theta_{i}\right)$$

Equation (5) is equal to formula (2) of the quantum model but with a completely different basement, endowed with a physiological connection.

**Figure 1:**
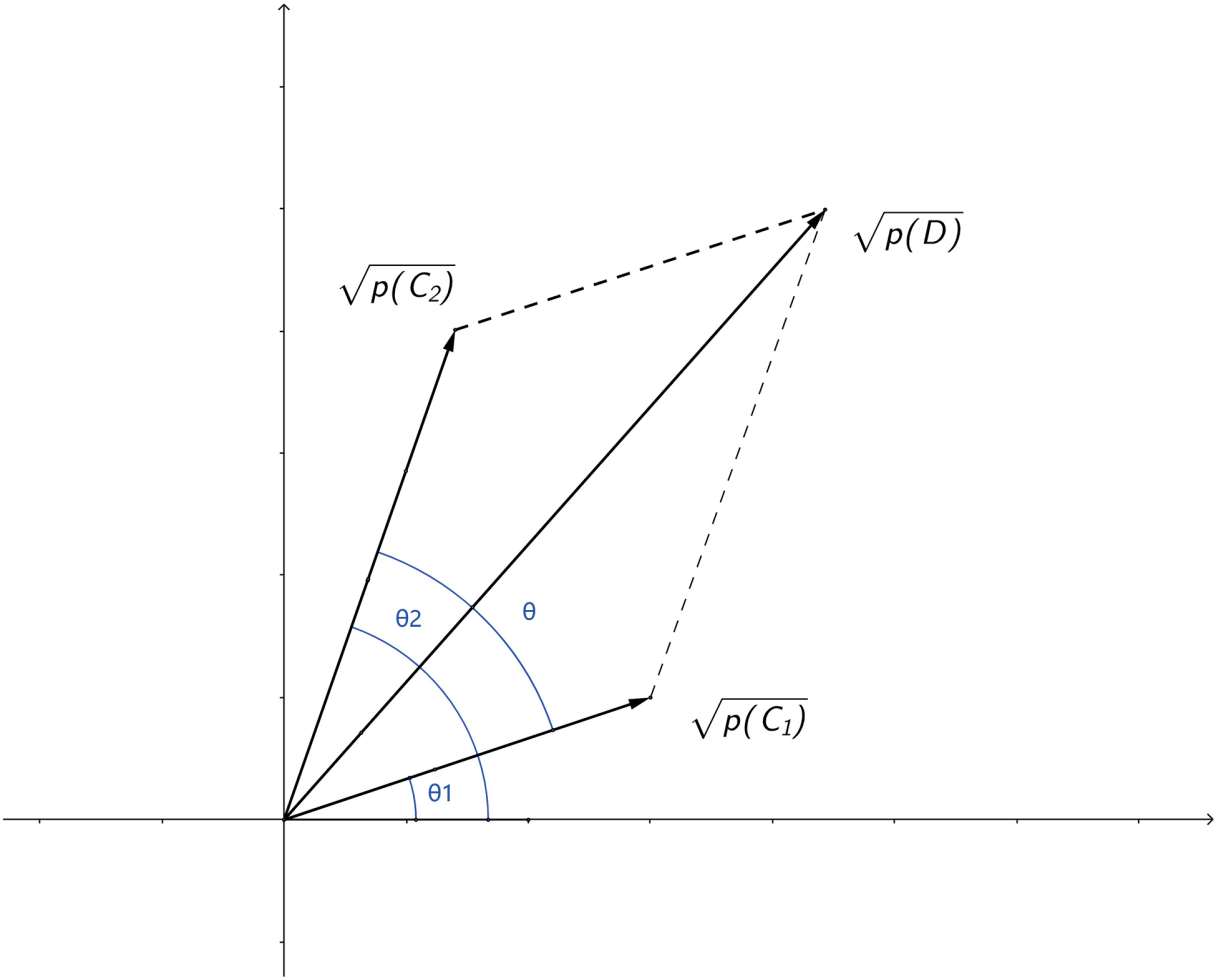
Relations of amplitudes (square roots of probabilities) visualized in vector form. Phase *θ* is the angle between the interfering amplitudes of conditions.

Comparing equation (3) and (5), there is an equivalence relation between the amplitudes and the square roots of the corresponding probabilities mathematically. Since amplitudes can be treated as vectors, the square roots of the corresponding probabilities are used to represent the amplitudes in vectors to facilitate the analysis of behavioral data. Therefore, the relation of amplitudes (square roots of probabilities) could be visualized as shown in Figure 1, where amplitudes (square roots of probabilities) are represented as vectors. The difference between phases of the two amplitudes is then mapped to the angle *θ* between the two vectors.

In reality, when people make decisions under uncertainty, they weigh the conditions and results repeatedly. Lacking a single mapping feature, they treat both the conditions and results as stimuli to define the decision, and the evoked waves will interfere with one another.

If decision *D* and condition *C* both have two states (*D^+^/D^−^* and *C^+^/C^−^*), and the probabilities of each state are *p(D^+^)*, *p(D^−^)*, *p(C^+^)* and *p(C^−^)*, respectively, the final decision will have four combinations: *D^+^C^+^*, *D^+^C^−^*, *D^−^C^+^*, and *D^−^C^−^*. Thus, formula (5) becomes


(6) $$\begin{array}{l} p(D^{+}C^{+})=p(D^{+})+p(C^{+})+2\sqrt{p(D^{+})\cdot p(C^{+})}\cos\theta_{1}\\ p(D^{+}C^{-})=p(D^{+})+p(C^{-})+2\sqrt{p(D^{+})\cdot p(C^{-})}\cos\theta_{2}\\ p(D^{-}C^{+})=p(D^{-})+p(C^{+})+2\sqrt{p(D^{-})\cdot p(C^{+})}\cos\theta_{3}\\ p(D^{-}C^{-})=p(D^{-})+p(C^{-})+2\sqrt{p(D^{-})\cdot p(C^{-})}\cos\theta_{4} \end{array}$$

where *θ_1_*, *θ_2_*, *θ_3_*, and *θ_4_* are the corresponding interference-phase differences.

Given *p(D^+^)*, *p(D^−^)*, *p(C^+^)* and *p(C^−^)*, *p(D^+^C^+^)*, *p(D^+^C^−^)*, *p(D^−^C^+^)* and *p(D^−^C^−^)* can have infinite mathematical solutions. However, after millions of years of evolution the *θ_1_*, *θ_2_*, *θ_3_*, and *θ_4_* of each decision should have settled in the human brain. Therefore, the probability of each combination should be relatively stable especially for people in the same cultural groups as Surov et al. (2019) found, and *p(D^+^C^+^)*, *p(D^+^C^−^)*, *p(D^−^C^+^)* and *p(D^−^C^−^)* each have unique solutions.

If subjects under the “unknown” condition know the empirical/theoretical value of *p(C^+^)* and *p(C^−^)* but not the exact condition, such as unknown the result of the first round in two-stage gambling task, they will first evaluate the probabilities of *C^+^* and *C^−^* as *p(C^+^)* and *p(C^−^)* or other values in their neighborhood, respectively. Then the brainwaves of *D^+^C^+^* and *D^+^C^−^* will interfere, and the probability of subjects making the *D*^+^ decision could be calculated by:


(7) $$\begin{array}{c} p(D^{+}|C\mathrm{-unknown})=p(D^{+}C^{+})+p(D^{+}C^{-})+\\ 2\sqrt{p(D^{+}C^{+})\cdot p(D^{+}C^{-})}\cdot \cos\theta \\ =p(C^{+})\cdot p(D^{+}|C^{+})+p(C^{-})\cdot p(D^{+}|C^{-})+\\ 2\sqrt{p(C^{+})\cdot p(D^{+}|C^{+})\cdot p(C^{-})\cdot p(D^{+}|C^{-})}\cdot \cos\theta \end{array}$$

where *p(D^+^|C^+^)* and *p(D^+^|C^−^)* also represent the empirical/theoretical probabilities.

Except for *θ*, all the parameters on the right side of equation (7) can be measured under the “known” condition. It is natural to suppose that *θ* is the function of *θ_1_*, *θ_2_*, *θ_3_*, and *θ_4_.* Mathematically, if the relationship between *θ* and *θ_1_*, *θ_2_*, *θ_3_* and *θ_4_* could be found, the probability of the “unknown” condition could be predicted.

Based on experiments in the literature, this study conducted a reverse-engineering process: First, calculate *θ_1_*, *θ_2_*, *θ_3_*, and *θ_4_* from formula (6) with the statistical probabilities of “known” conditions in the experiment. Then, derive the experimental cos*θ*, defined as cos*θ_expt_*, from formula (7) with the observed probabilities of “unknown” condition in the experiment:


(8) $$cos\theta_{expt}=\frac{p\left(D^{+}|C-unknown\right)-p\left(D^{+}C^{+}\right)-p\left(D^{+}C^{-}\right)}{2\sqrt{p\left(D^{+}C^{+}\right)•p\left(D^{+}C^{-}\right)}}$$

Finally, the relationship between *θ_expt_* and *θ_1_*, *θ_2_*, *θ_3_*, and *θ_4_* can be analyzed. The estimation of *θ*, defined as *θ_fit_*, could be described as


(9) $$\theta_{fit}=F\left(\theta_{1},\theta_{2},\theta_{3},\theta_{4}\right)$$

Now, with *θ_fit_* and observed probabilities of “known” conditions in the experiments, one is able to predict the corresponding probability under “unknown” conditions from formula (7).

## Empirical Test

### Data

#### Event-Related Potential Data

The primary work of verifying the proposed model is to test whether interference really happens between brainwaves in the decision-making process. Given the status of electrophysiological research to date, the exact intensity of brainwaves cannot be measured. EEG and MEG only partially describe the whole picture of brainwaves. Fortunately, the phase of a brainwave should be stable no matter how it attenuates during transmission or detection, which gives an alternative way to test the hypothesis of brainwave interference. During decision making, the valence of the stimulus will be classified into three categories (positive, negative, neutral), but from the perspective of structure and energy consumption optimization there should be only two scales in the brain: positive (e.g., 1, 2, X) and negative (e.g., −1, −2, −X), with the neutral expressed as the interference of the former two. Therefore, one of the test methods is to investigate the brainwaves of the positive, negative and neutral conditions to ascertain whether the brainwave of the neutral condition represents the interference of the brainwaves of the other two conditions.

Although it cannot be assumed that electroencephalographic waves are exact copies of brainwaves in the human brain, this study use ERP to represent the brainwaves and treats the voltage amplitude as the amplitude of the waves. The intensity of the brainwave with a certain stimulus may vary at different cerebral cortex locations, but the interference-phase difference should be stable across all locations when it interacts with another brainwave. ERP data are abundant in literature, but it is rare to find experiments involving all three kinds of valences. Following three experiments are available:

Experiment A: Mao and Wang (2011) recorded the ERP N270 while participants performed a dual-attribute matching task under different attentive conditions. Visual stimulus spots were sequentially presented in pair of four categories: same color and position; same color but different position; same position but different color; and different color and position. Participants were instructed to discriminate whether the attributes of the first stimulus were the same as those of the second with different attention instructions: attending to color and attending to position, yielding three categories: task-irrelevant conflict (the conflict was irrelevant to the attention task, namely, the color was different when attending to position), task-relevant conflict, and conjunction conflicts (both the color and position were different in both kinds of attention task). Table 1 shows the average N270 amplitudes of each category.

**Table 1:** Average amplitudes of N270 under different conflict conditions of color and position and interference phase differences.

Electrode location	Task-relevant conflict	Task-irrelevant conflict	Conjunction conflicts	*θ*
F7	7.93	4.6	5.19	141.26°
F8	8.42	5.13	6.45	129.79°
T5	5.66	2.69	3.61	149.32°
T6	5.18	2.24	3.36	152.87°

Columns 2–4 show the average amplitudes (unit: μν) of N270 in the experiments of Mao and Wang (2011), column 5 are the computed interference-phase differences.

Experiment B: Suo et al. (2012) recorded the ERPs of participants engaged in a slot gambling task to investigate how the closeness of outcome influenced evaluation of the win. Table 2 lists the average N300 amplitudes under different outcome distances (near, medium, and far).

**Table 2:** Average amplitudes of P300 under different outcome distances and interference phase differences.

Electrode location	Far	Near	Medium	*θ*
Fz	20.93	14.94	16.29	129.05°
FCz	22.91	16.95	18.29	128.32°
Cz	23.88	18.83	20.23	124.75°

Columns 2–4 show the average amplitudes (unit: μν) of P300 in the experiments of Suo et al. (2012), column 5 are the computed interference-phase differences.

Experiment C: Wang et al. (2014a) recorded participants’ ERP while they watched photos depicting two actors’ actions and performed an intention inference task. Three action intentions were involved: kindness, hostility and non-interactiveness. Table 3 presents the average P300 amplitudes.

**Table 3:** Average amplitudes of P300 under different action intentions and interference phase differences.

Electrode location	Kind	Hostile	Non-interactive	*θ*
Central zero	8.86	12.11	10.31	123.37°
Right part	9.95	12.38	10.36	126.16°
Central zone	8.35	10.91	9.41	123.36°
Parietal zone	11.72	15.49	12.54	127.59°

Columns 2–4 show the average amplitudes (unit: μν) of P300 in the experiments of Wang et al. (2014a), column 5 are the computed interference-phase differences.

The scalp electrodes in above experiments were placed following the international 10–20 system.

#### Categorization-Decision Experiment Data

Townsend et al. (2000), Busemeyer et al. (2009), and Wang and Busemeyer (2016) successively conducted categorization-decision experiments with similar methods. On each trial, participants were shown pictures of faces that varied in facial width. Participants were asked to categorize the faces as belonging to either a “good” guy or “bad” guy and/or were asked to decide whether to attack or withdraw. In the C-then-D condition, participants made a categorization followed by an action decision, and in the D-alone condition, participants made only an action decision. The researchers found that the law of total probability was nearly satisfied for the wide faces but was violated for the narrow faces. According to the law of total probability, the probability of attack in the D-alone condition should within the probabilities of attack when the face was categorize to “good” [p(attack|good)] and “bad” [p(attack|bad)] in the C-then-D condition. However, the experiment shown that the proportion of attack actions in the D-alone condition was higher than both p(attack|good) and p(attack|bad) for narrow faces (Table 4).

**Table 4:** Categorization-decision experiments results in literature and model fitting.

Literature	*Face type*	*p(attack|good)*	*p(attack|bad)*	*p(good)*	*p(bad)*	*p(attack|D-Alone)*	*θ_expt_*	*θ_1_*	*θ_2_*	*θ_3_*	*θ_4_*	*θ_fit_*	*P_fit_*
Townsend et al., 2000	W	0.35	0.52	0.84	0.16	0.39	87.65°	129.33°	145.09°	180.00°	157.52°	85.58°	0.40
	N	0.41	0.63	0.17	0.83	0.69	75.22°	155.02°	180.00°	143.12°	129.90°	75.08°	0.69
Busemeyer et al., 2009	W	0.37	0.53	0.8	0.2	0.39	91.94°	130.26°	143.02°	180.00°	151.00°	86.72°	0.42
	N	0.45	0.64	0.2	0.8	0.69	78.17°	149.86°	180.00°	143.74°	129.88°	80.25°	0.67
Wang and Busemeyer, 2016 Experiment 1	W	0.39	0.52	0.78	0.22	0.42	89.79°	131.11°	141.50°	166.60°	149.73°	87.39°	0.44
	N	0.41	0.58	0.21	0.79	0.59	83.39°	151.13°	171.19°	139.60°	131.91°	76.96°	0.63
Wang and Busemeyer, 2016 Experiment 2	W	0.33	0.53	0.78	0.22	0.37	90.66°	129.21°	146.10°	180.00°	146.32°	84.69°	0.41
	N	0.37	0.61	0.24	0.76	0.6	83.26°	144.91°	165.06°	141.37°	130.92°	84.17°	0.59
Wang and Busemeyer, 2016 Experiment 3	W	0.34	0.58	0.77	0.23	0.39	90.80°	129.42°	144.97°	171.74°	144.65°	85.62°	0.42
	N	0.33	0.66	0.24	0.76	0.62	84.36°	141.78°	173.30°	144.65°	129.17°	89.05°	0.59

Columns 3–7 show the observed probabilities in the experiments. *θ_expt_*, *θ_1_*, *θ_2_*, *θ_3_*, and *θ_4_* are the computed interference-phase differences of *p(attack|D-Alone)*, *p(withdraw|good)*, *p(attack|good)*, *p(withdraw|bad)*, and *p(attack|good)*, respectively, from observed probabilities. *θ* = 180° when *cosθ* is slightly <−1 due to random error. *θ_fit_* is the fit value of *θ_expt_* by BIDM model. *p_fit_* is the *p(attack|D-alone)* fitted by BIDM model with *θ_fit_*.

#### Two-Stage Gambling Experiment Data

Tversky and Shafir (1992), Kühberger et al. (2001), Lambdin and Burdsal (2007), and Surov et al. (2019) studied two-stage gambling experiments in which participants were asked at each stage to decide whether or not to replay a gamble with an equal chance of winning or losing if they had either won, lost or did not know the outcome of the first gamble. The results revealed that some participants decided to play again no matter they had won or lost the first gamble. Through Savage’s sure-thing principle, those participants were expected to choose to play again even if they did not know the outcome of the first one. And according to the law of total probability, the probability of choosing to play the second gamble when the outcome of the first one was unknown [p(again|unknown)] should within the minimum and maximum values of the probability of deciding to play again while won [p(again|win)] or lost [p(again|lose)] the first gamble. However, it came out that only a part of them (less than the minimum probability) decided to play the second gamble (Table 5), violating the law of total probability and the sure-thing principle.

**Table 5:** Results of two-stage gambling experiments in literature and model fitting.

Literature	*p(again|win)*	*p(again|lose)*	*p(win)*	*p(lose)*	*p(again|unknown)*	*θ_expt_*	*θ_1_*	*θ_2_*	*θ_3_*	*θ_4_*	*θ_fit_*	*p_fit_*
Tversky and Shafir, 1992 Experiment 1	0.69	0.57	0.5	0.5	0.38	113.49°	134.37°	146.22°	138.83°	139.59°	106.89°	0.45
Tversky and Shafir, 1992 Experiment 2	0.69	0.59	0.5	0.5	0.35	117.03°	134.64°	146.19°	138.32°	140.53°	106.91°	0.4
Tversky and Shafir, 1992 Experiment 3	0.71	0.56	0.5	0.5	0.84	71.03°	133.80°	147.43°	139.35°	139.02°	106.29°	0.46
Kühberger et al., 2001Experiment 1	0.6	0.47	0.5	0.5	0.47	97.03°	135.28°	142.49°	140.66°	136.54°	108.75°	0.36
Kühberger et al., 2001 Experiment 2	0.83	0.7	0.5	0.5	0.62	100.97°	133.41°	161.46°	137.71°	148.57°	99.27°	0.64
Kühberger et al., 2001 Experiment 3	0.8	0.37	0.5	0.5	0.43	106.55°	129.29°	153.45°	146.31°	131.19°	103.27°	0.46
Kühberger et al., 2001 Experiment 4	0.68	0.32	0.5	0.5	0.38	104.91°	131.30°	147.14°	147.14°	131.30°	106.43°	0.37
Lambdin and Burdsal, 2007	0.64	0.47	0.5	0.5	0.38	108.61°	134.24°	144.18°	141.11°	136.12°	107.91°	0.39
Surov et al., 2019	0.3	0.24	0.5	0.5	0.17	111.88°	147.53°	136.74°	152.19°	134.71°	111.63°	0.17

Columns 2–6 show the observed probabilities in the experiments. *θ_expt_*, *θ_1_*, *θ_2_*, *θ_3_*, and *θ_4_* are the computed interference-phase differences of *p(again|unknown)*, *p(again|win)*, *p(stop|win)*, *p(again|lose)*, and *p(stop|win)*, respectively, from observed probabilities. *θ_fit_* is the fit value of *θ_expt_* by BIDM model. *p_fit_* is the *p(again|unknown)* fitted by BIDM model with *θ_fit_*.

#### Data of the Prisoner’s Dilemma Experiments

Li and Taplin (2002) faced violation to the law of total probability and the sure-thing principle in their prisoner’s dilemma experiments. This game consisted of two players who were separately confined and could not communicate with each other. The researchers tested three conditions in the prisoner’s dilemma game: the player knew that the other defected (k-d), the player knew that the other cooperated (k-c), or the player did not know the other player’s action (unknown). The last condition showed a deviation from the classical probability theory, suggesting that a significant percentage of players did not act according to the law of total probability (Table 6).

**Table 6:** Experiments results of the prisoner’s dilemma game and model fitting.

Experiment	*p(defect|k-d)*	*p(defect|k-c)*	*p(k-d)*	*p(k-c)*	*p(defect|unknown)*	*θexpt*	*θ_1_*	*θ_2_*	*θ_3_*	*θ_4_*	*θ_fit_*	*p_fit_*
1	0.73	0.67	0.50	0.50	0.60	98.23°	134.78°	149.39°	137.09°	144.87°	105.30°	0.52
2	0.80	0.77	0.50	0.50	0.63	101.29°	134.89°	159.52°	135.97°	155.71°	100.24°	0.64
3	0.90	0.87	0.50	0.50	0.87	91.08°	134.60°	180.00°	135.62°	180.00°	90.00°	0.88
4	0.83	0.8	0.50	0.50	0.70	98.21°	134.77°	172.23°	135.83°	164.44°	93.88°	0.76
5	0.83	0.73	0.50	0.50	0.70	96.12°	133.82°	164.17°	137.08°	152.39°	97.92°	0.68
6	0.77	0.83	0.50	0.50	0.80	90.00°	136.44°	157.27°	134.29°	167.16°	101.36°	0.64
7	0.87	0.73	0.50	0.50	0.77	92.39 °	133.25°	180.00°	137.55°	153.63°	90.00°	0.80

Experiments 1–7: Li and Taplin (2002) experiments 1–7. Columns 2–6 show the observed probabilities in the experiments. *θ_expt_*, *θ_1_*, *θ_2_*, *θ_3_*, and *θ_4_* are the computed interference-phase differences of *p(defect|unknown)*, *p(defect|k-d)*, *p(defect|k-c)*, *p(collaborate|k-d)*, and *p(collaborate|k-c)*, respectively, from observed probabilities. *θ* = 180° when *cosθ* is slightly <−1 due to random error. *θ_fit_* is the fit value of *θ_expt_* by BIDM model. *p_fit_* is the *p(defect|unknown)* fitted by BIDM model with *θ_fit_*.

### Results

#### Brainwave Interference Test

As stated above (Event-Related Potential Data), it is implicitly assumed that a brainwave evoked by a neutral stimulus could be treated as the interference of the brainwaves produced by the positive and negative stimuli. From formula (5), the following equation is obtained and used to define the interference-phase difference *θ*:


(10) $$I_{neutral}=I_{positive}+I_{negative}+2\cdot \sqrt{I_{positive}I_{negative}}\;\cos\theta$$

In experiment A, the brainwave of the conjunction conflict is the interference result of the brainwaves of the task-irrelevant and task-relevant conflicts. The calculation shows that the interference-phase differences are relatively stable with a lower coefficient of variation (*CV* = 0.07) than that of the amplitudes (*CV* = 0.24 ~ 0.39; Table 1).

In experiment B, the brainwave when the outcome distance is moderate counted as the interference result of brainwaves when the outcome distances are near and far. The calculation also showed that the interference-phase differences are more stable (*CV* = 0.02) than that of the amplitudes (*CV* = 0.07 ~ 0.12; Table 2).

In experiment C, the brainwave of non-interactive action is treated as the interference result of brainwaves of kind and hostile actions. Again, the interference-phase differences are almost constant (*CV* = 0.02) compared with that of the amplitudes (*CV* = 0.13 ~ 0.15; Table 3).

**Figure 2:**
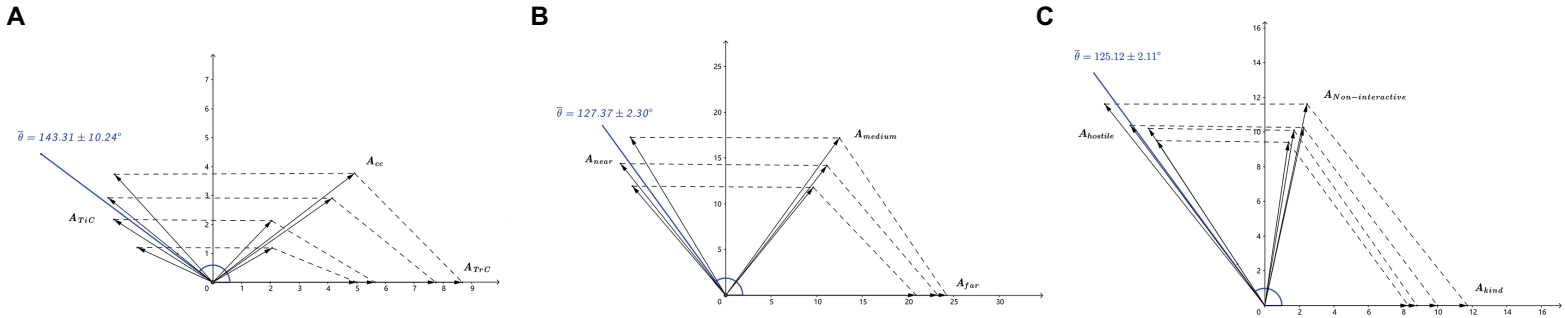
Verification of the interference of brainwave. **(A)** Experiment A (Mao and Wang, 2011). *A_TiC_*, *A_TrC_*, and *A_CC_* represent the amplitudes of task-irrelevant conflict, task-relevant conflict, and conjunction conflicts, respectively. **(B)** Experiment B (Suo et al., 2012). *A_far_*, *A_near_*, and *A_medium_* represent the amplitudes under far, near, and medium outcome distances, respectively. **(C)** Experiment C (Wang et al., 2014a). *A_kind_*, *A_hostile_*, and *A_non-interactive_* represent the amplitudes under kindness, hostility and non-interactiveness action intentions, respectively. Phase *θ* are the angles between the interfering amplitudes of conditions.

These results support the hypothesis that when two stimulus inputs are involved in the decision, the brainwaves triggered by both may interfere and lead to the decision (Figure 2), laying the physiological (psychological) foundation for the brainwave-interference model.

#### Test With Categorization-Decision Experimental Data

Table 4 presents the interference-phase differences *θ_1_*, *θ_2_*, *θ_3_*, and *θ_4_* calculated from equation (6) using the measured probabilities in the experiments. They are stable under the same face types (wide face: *θ_1_* = 129.87 ± 0.81°, *θ_2_* = 144.14 ± 1.85°, *θ_3_* = 175.67 ± 6.20°, *θ_4_* = 149.84 ± 4.99°; narrow face: *θ_1_* = 148.54 ± 5.23°, *θ_2_* = 173.91 ± 6.33°, *θ_3_* = 142.50 ± 2.01°, *θ_4_* = 130.36 ± 1.07°).

**Figure 3:**
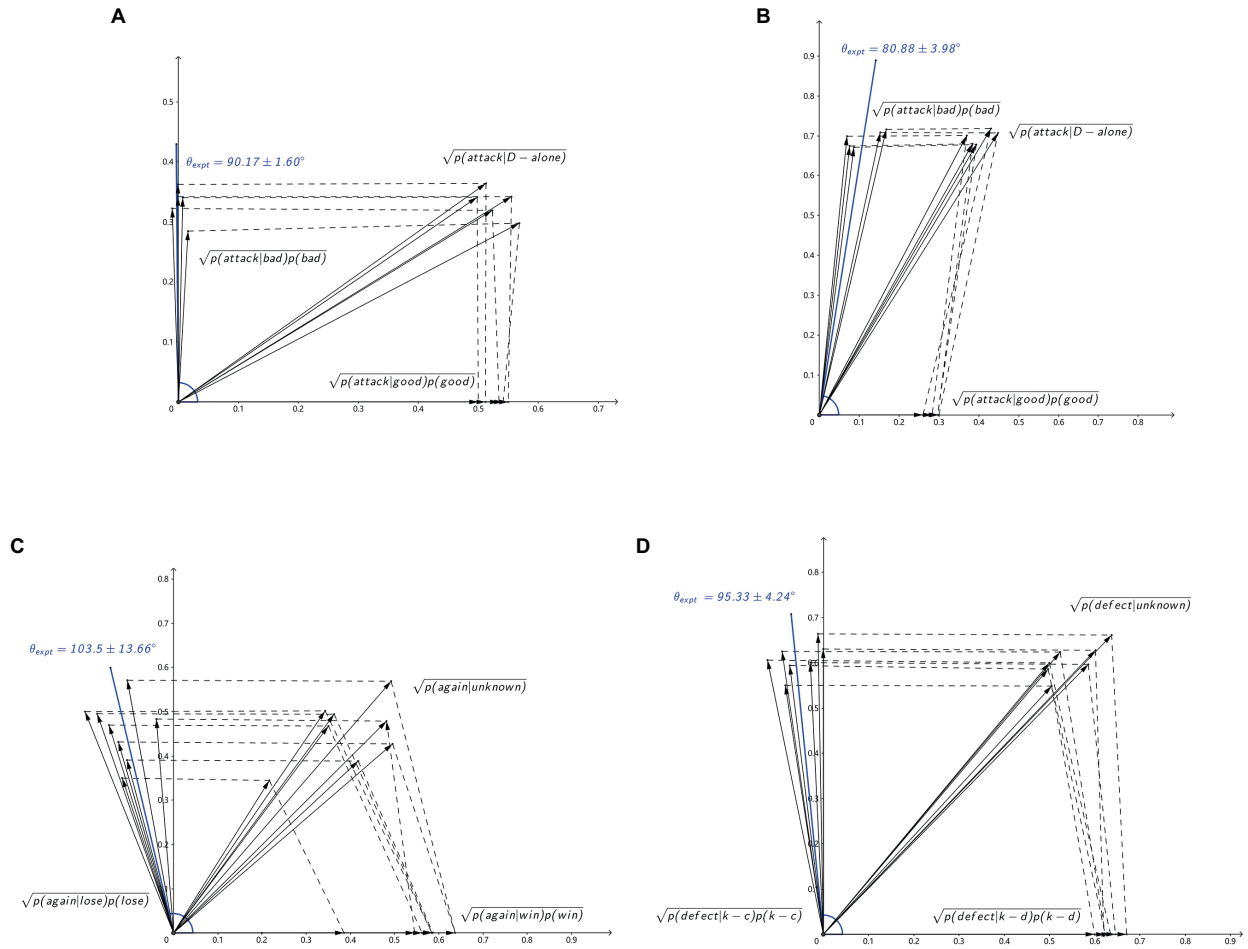
Verification of the interference-phase difference stability. **(A)** Categorization-decision experiment (wide face), **(B)** categorization-decision experiment (wide face), **(C)** two-stage gambling experiment, **(D)** prisoner’s dilemma experiment. Probabilities of the relevant experiments in vector amplitude form are shown in black. Interference-phase difference *θ_expt_* of “unknown” conditions computed with brainwave-interference decision model (BIDM) are shown in blue.


After applying the measured probabilities *p(attack|D-alone)*, *p(good)*, *p(bad)*, *p(attack|good)*, *p(attack|bad)* to formula (7), *θ_expt_* is obtained (Table 4; wide face: *θ_expt_* = 90.17 ± 1.60°, Figure 3A; narrow face: *θ_expt_* = 80.88 ± 3.98°, Figure 3B). Fitting *θ_expt_* with *θ_1_*, *θ_2_*, *θ_3_*, and *θ_4_*, *θ_fit_* is given by

(11) $$\theta_{fit}=2\pi -\left(\theta_{1}+\theta_{2}\right)\;$$

for the wide-face experiments (*θ_fit_* = 86.00 ± 1.06°, RMSD = 4.67°), where *θ_1_* and *θ_2_* are the interference-phase differences of *p(withdraw|good)* and *p(attack|good)*, respectively, and.


(12) $$\theta_{fit}=2\pi -\left(\theta_{1}+\theta_{4}\right)$$

for the narrow-face experiments (*θ_fit_* = 81.10 ± 5.63°, RMSD = 3.70°), where *θ_1_* and *θ_2_* are the interference-phase differences of *p(withdraw|good)* and *p(attack|bad)*, respectively.

Then, *p(attack|D-alone)* for wide face could be estimated as


(13) $$\begin{array}{c} p_{fit}\left(attack|D-alone\right)=p\left(good\right)\cdot p\left(attack|good\right)+p\left(bad\right)\cdot p\left(attack|bad\right)\\ +2\cdot \sqrt{p\left(good\right)\cdot p\left(attack|good\right)\cdot p\left(bad\right)\cdot p\left(attack|bad\right)}\cdot \cos\left(\theta_{1}+\theta_{2}\right) \end{array}$$

and *p(attack|D-alone)* for narrow face could be estimated as


(14) $$\begin{array}{c} p_{fit}\left(attack|D-\mathrm{alone}\right)=p\left(good\right)\cdot p\left(attack|good\right)+p\left(bad\right)\cdot p\left(attack|bad\right)\\ +2\cdot \sqrt{p\left(good\right)\cdot p\left(attack|good\right)\cdot p\left(bad\right)\cdot p\left(attack|bad\right)}\cdot \cos\left(\theta_{1}+\theta_{4}\right) \end{array}$$

The estimated probabilities of both equations could fit the observed results with a minor mean absolute percentage error (MAPE; for wide face: MAPE = 0.06; for narrow face: MAPE = 0.03).

#### Test With Two-Stage Gambling Experimental Data

The interference-phase differences obtained from equation (6) are relatively stable (*θ_1_* = 134.87 ± 5.09°, *θ_2_* = 147.26 ± 6.94°, *θ_3_* = 142.40 ± 4.99°, *θ_4_* = 137.51 ± 5.33°; Table 5). The *θ_expt_* for *p(again|unknown)* calculated from formula (7) is 103.5 ± 13.66° (Figure 3C). Fitting *θ_expt_* with *θ_1_*, *θ_2_*, *θ_3_*, and *θ_4_*, *θ_fit_* is given by


(15) $$\theta_{fit}=\frac{2\pi -\theta_{2}}{2}$$

where *θ_2_* is the interference-phase difference of *p(not play|win)*. *θ_fit_* is 106.37 ± 3.47° (RMSD = 13.09°).

Then, *p(again|unknown)* could be estimated by


(16) $$\begin{array}{c} p_{fit}\left(again|unknown\right)=p\left(win\right)\cdot p\left(again|w\mathrm{in}\right)+p\left(lose\right)\cdot p\left(again|l\mathrm{ose}\right)\\ +2\cdot \sqrt{p\left(win\right)\cdot p\left(again|w\mathrm{in}\right)\cdot p\left(lose\right)\cdot p\left(again|l\mathrm{ose}\right)}\cdot \cos\left(\frac{2\pi -\theta_{2}}{2}\right) \end{array}$$

The *p_fit_(again|unknown)* fits the real data (Table 5). Only experiment 3 of Tversky and Shafir (1992) deviated from this trend. All three decision tasks in this experiment were presented on the same instruction page, which presumably aimed to encourage subjects toward rationally consistent decision making. This led to a much higher *p(again|unknown)* than that of the other experiments, in which decisions were separated in time or assigned to different subjects. Experiment 3 of Kühberger et al. (2001) used the same method as the experiment 3 of Tversky and Shafir (1992), and after excluding these two experiments, *θ_fit_* is 106.83 ± 3.77° (RMSD = 6.12°) and the fitting MAPE of the probabilities of remaining experiments is 0.06.

#### Test With Prisoner’s Dilemma Experimental Data

The interference-phase differences obtained from equation (6) were relatively stable (*θ_1_* = 134.65 ± 1.00°, *θ_2_* = 166.08 ± 11.75°, *θ_3_* = 136.20 ± 1.12°, *θ_4_* = 159.74 ± 11.67°; Table 6).

The *θ_expt_* for *p(defect|unknown)* obtained from formula (7) is 95.33 ± 4.24° (Figure 3D). Fitting *θ_expt_* with *θ_1_*, *θ_2_*, *θ_3_*, and *θ_4_*, *θ_fit_* is given by


(17) $$\theta_{fit}=\frac{2\pi -\theta_{2}}{2}$$

where *θ_2_* is the interference-phase difference of *p(defect|k-c)*. *θ_fit_* is 96.96 ± 5.87° (RMSD = 5.46°).

Then, *p(defect|unknown)* can be estimated by the following equation with MAPE of 0.06.


(18) $$\begin{array}{c} p_{\mathrm{fit}}\left(defect|unknown\right)=p\left(k-d\right)\cdot p\left(defect|k-d\right)+p\left(k-c\right)\cdot p\left(defect|k-c\right)\\ +2\cdot \sqrt{p\left(k-d\right)\cdot p\left(defect|k-d\right)\cdot p\left(k-c\right)\cdot p\left(defect|k-c\right)}\cdot \cos\left(\frac{2\pi -\theta_{2}}{2}\right) \end{array}$$

## Discussion

### Phase Stability

**Figure 4:**
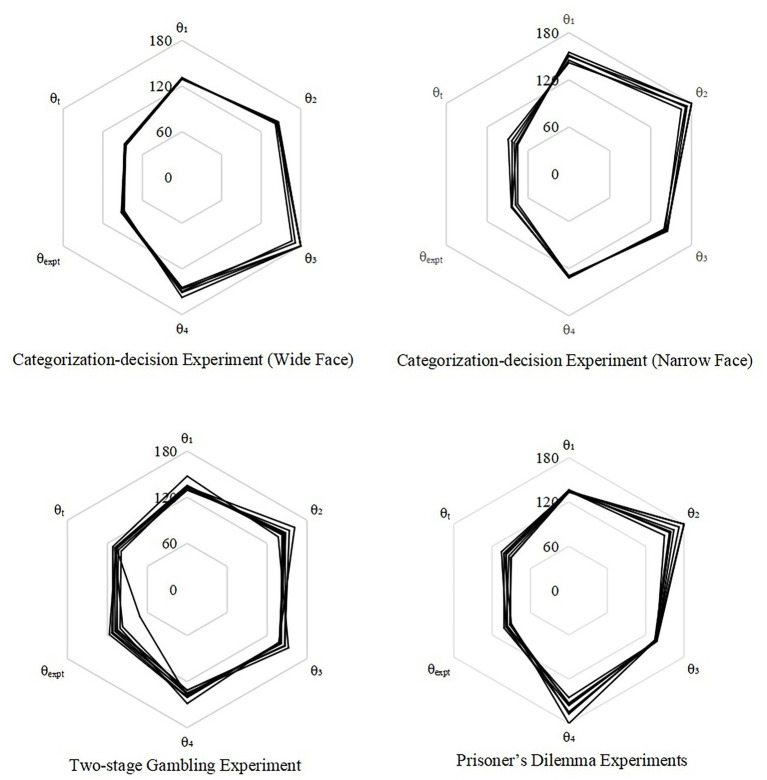
Phase stability in different kinds of behavior experiments. Grids represent the value of phase from 0° to 180°. *θ_1_*, *θ_2_*, *θ_3_*, *θ_4_*, and *θ_expt_* are the computed interference-phase differences from observed probabilities in each experiment. *θ_t_* is the fitted value of *θ_expt_* by BIDM model. The closeness of the lines at each axis indicates the stability.

In all these three behavior experiments mentioned above, *θ_1_*, *θ_2_*, *θ_3_* and *θ_4_* remained relatively stable (Figure 4). The SD of their distribution from the mean was 1°–18°. In reality, the probabilities of five random numbers from the uniform distribution on the interval [0°, 180°] falling into window of 2° and 36° are <1/59000000000 and <1/3125, respectively. This compactness of the phase distribution was not a coincidence but a regularity, thus supporting the hypothesis that evolution has settled the phase in the human brain.

However, this stability is relatively and culturally specific. In the two-stage gambling experiments, the phase of the last experiment was obviously distinct (Table 5). For example, *θ_1_* was ~134*°* for subjects from the US (Tversky and Shafir, 1992; Lambdin and Burdsal, 2007) and Austria (Kühberger et al., 2001) but was 147*°* for subjects from Russia (Surov et al., 2019), possibly due to the diversity of risk appetite in different cultures. Nevertheless, the BIDM model can precisely predicted the disjunction effect, regardless of phase variation across cultures.

In the categorization-decision experiment, an interesting symmetrical relationship occurred in the interference phase difference of each experimental pair (Table 4), which was similar to the QQ equality in the question order effect proposed by Wang et al. (2014b). *θ_1_* of the narrow faces approximately equaled *θ_4_* of the wide faces, and *θ_2_* of the narrow faces was close to *θ_3_* of the wide faces and vice versa.

Moreover, the sum of *θ_1_* and *θ_2_* was close to the sum of *θ_3_* and *θ_4_* in both the two-stage gambling experiments and the prisoner’s dilemma experiments. This may caused by the 50/50 chance of winning/losing and other player defect/collaborate.

### Unification of the Interference Phase of an Unknown Condition

Surov et al. (2019) directly obtained phase *θ* of an unknown condition from the quantum model, missing the relationship between the phases of the different conditions of known and unknown. The BIDM model bridges the phases *θ_1_*, *θ_2_*, *θ_3_* and *θ_4_* with *θ*, meaning that the decision mechanism in the unknown condition is not a new one but the synthesis of the decision mechanism under the known condition. Surov et al. (2021) combined the four individual phases to a single four-phase difference as a fitting parameter allowing to tune concurrence value in the quantum model, similar in form of *θ* = *θ_1_* + *θ_2_*−*θ_3_*−*θ_4_*, which is totally different from their functional relation and the implicit interference logic in BIDM.

Their relationship appears to have regularity for various behaviors. For categorization-decision behavior, *θ_fit_* is defined by the phases of the safest and riskiest choices. Wide faces were most likely to be labeled as good; thus, *p(withdraw|good)* and *p(attack|good)* were the most reliable and risky choices, with the maximum and minimum probabilities among those four combination of conditions and decisions in the experiments, respectively, and their phases *θ_1_* and *θ_4_* determined *θ* (equation 13). Narrow faces had the opposite result (equation 14). For disjunction effects in both the two-stage gambling and prisoner’s dilemma, *θ_fit_* was defined by the phase of the highest risky choices with the minimum probability in the experiments *[p(not play|win)* and *p(defect|k-c) respectively]*. Therefore, the function of *θ_fit_* and *θ_1_*, *θ_2_*, *θ_3_*, *θ_4_* could be integrated as


(19) $$\theta_{fit}=2\pi -\left(\theta_{\mathrm{safest}}+\theta_{riskiest}\right)$$

for categorization-decision behaviors (RMSD = 4.10°), and


(20) $$\theta_{fit}=\frac{2\pi -\theta_{riskiest}}{2}$$

for disjunction effect behaviors (RMSD = 5.96°), where *θ_safest_* and *θ_riskiest_* are the phases of the safest and riskiest choices, respectively.

Both (19) and (20) are involving the riskiest choice, revealing that humans mainly show aversion to risk under unknown conditions.

### Predictive Power Comparison

**Figure 5:**
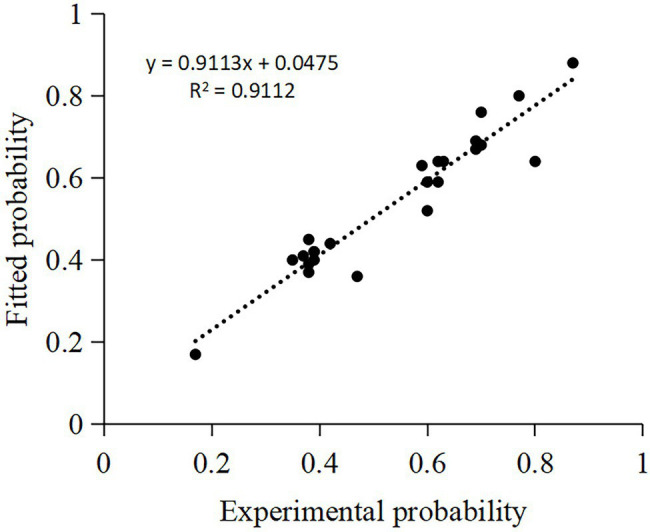
Fitting quality of the BIDM model. *X*-axis represents the probabilities under “unknown” conditions in the categorization-decision experiment, two-stage gambling experiment, and prisoner’s dilemma experiment, and *Y*-axis represents the probabilities fitted with BIDM model.

**Figure 6:**
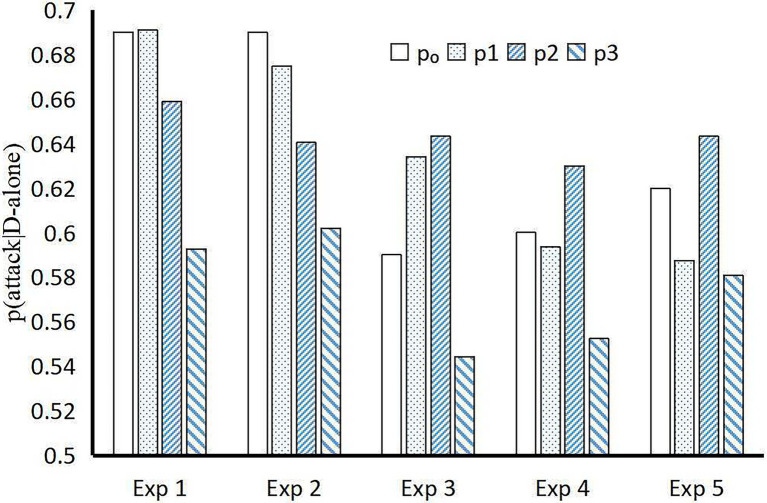
Predicted disjunction effect of different models in categorization-decisions. *p*_0_ is the *p(attack|D-alone)* observed in experiments; *p*_1_, *p*_2_, and *p*_3_ are the predictions of BIDM model, evidential Markov model (He and Jiang, 2018a), and the classical law of total probability, respectively. Exp. 1–5: experiments regarding narrow-face types in Townsend et al. (2000), Busemeyer et al. (2009), and Wang and Busemeyer (2016), experiments 1–3.

BIDM model make it convenient and precisely to predict the probability under “unknown” conditions with probabilities under “known” conditions (Figure 5). He and Jiang (2018a) proposed an evidential Markov (EM) model to predict the disjunction effect in categorization-decision experiments, which has fewer free parameters than do the seven existing models. However, the BIDM model fits the experiments results more accurate (MAPE = 0.03) than the EM model (MAPE = 0.06; Figure 6) with merely one free parameter.

**Figure 7:**
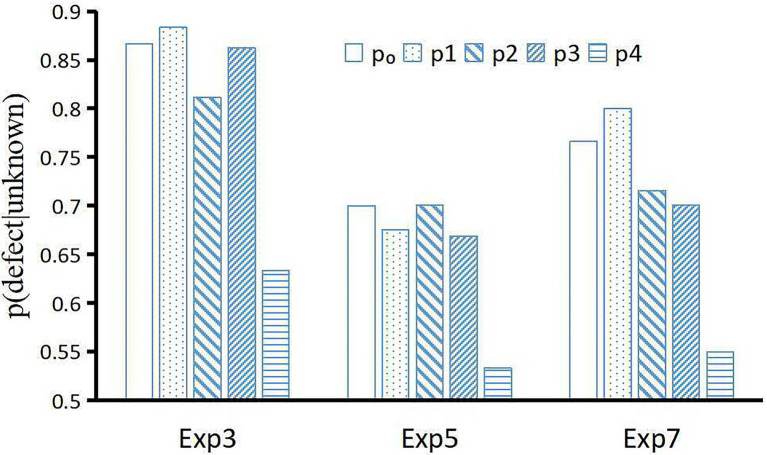
Predicted disjunction effect of different models in the prisoner’s dilemma game. Huang et al. (2019) only compared experiments 3, 5, and 7 of Li and Taplin (2002). *p*_0_ is the *p(defect|unknown)* observed in the experiments, *p*_1_, *p*_2_, *p*_3_, *p*_4_ are predicted by the BIDM model, Moreira and Wichert (2016), Huang et al. (2019), Yukalov and Sornette (2011), respectively.

Huang et al. (2019) proposed a new quantum-like Bayesian network model to accommodate the paradoxical phenomenon in the prisoner’s dilemma game; its predictive power was better than that of the quantum prospect decision theory (Yukalov and Sornette, 2011) and quantum-like Bayesian network model (Moreira and Wichert, 2016). However, Figure 7 shows that the fitting accuracy of the BIDM model is comparable (MAPE = 0.04) with the models of Moreira and Wichert (2016; MAPE = 0.03) and Huang et al. (2019; MAPE = 0.04).

This study, however, is subject to several limitations, mainly concerning the data. First, applicable experiment recording both the neurophysiological and behavioral data is not available, and this study has to divide the verification into two separate parts, weakening the linkage between neural activity and behavior. Second, the results are based on small data set. Third, existing experimental data are used without scrutiny of their contexts, which may increase the risk of bias. Although, to some extent, this demonstrates the robustness of the model from another point of view.

## Conclusion

The proposed brainwave-interference decision-making model is applicable in both neurophysiological and decision-making analysis. The hypothesis of brainwave interference is verified by psychological experimental data. Results of applying the proposed model in behavior experimental data show that the interference-phase differences under “unknown” conditions are derived from corresponding interference-phase differences of the highest-risk choices under “known” conditions, and all those interference-phase differences are stable across subject backgrounds. Testing the predictive power of brainwave-interference decision-making model in other kinds of human behavior with much larger set of data, and detailed analysis of psychological meaning of the phase of the brainwave and the carrier/transmission of the brainwave, are some of the subjects for future study.

## Data Availability Statement

The original contributions presented in the study are included in the article/supplementary material, further inquiries can be directed to the corresponding author.

## Ethics Statement

Ethical review and approval was not required for the study on human participants in accordance with the local legislation and institutional requirements. Written informed consent for participation was not required for this study in accordance with the national legislation and the institutional requirements.

## Author Contributions

Z-HS conceived the study, performed the analysis, and wrote the manuscript.

## Conflict of Interest

The author declares that the research was conducted in the absence of any commercial or financial relationships that could be construed as a potential conflict of interest.

## Publisher’s Note

All claims expressed in this article are solely those of the authors and do not necessarily represent those of their affiliated organizations, or those of the publisher, the editors and the reviewers. Any product that may be evaluated in this article, or claim that may be made by its manufacturer, is not guaranteed or endorsed by the publisher.

## References

[ref1] Barros J. (2012). Quantum-like model of behavioral response computation using neural oscillators. Biosystems 110, 171–182. doi: 10.1016/j.biosystems.2012.10.002, PMID: 23127789

[ref2] Barros J. Suppes P. (2009). Quantum mechanics, interference, and the brain. J. Math. Psychol. 53, 306–313. doi: 10.1016/j.jmp.2009.03.005

[ref3] Bode S. Feuerriegel D. Bennett D. Alday P. M. (2019). The decision decoding ToolBOX (DDTBOX)–A multivariate pattern analysis toolbox for event-related potentials. Neuroinformatics 17, 27–42. doi: 10.1007/s12021-018-9375-z, PMID: 29721680 PMC6394452

[ref4] Broekaert J. B. Busemeyer J. R. Pothos E. M. (2020). The disjunction effect in two-stage simulated gambles. An experimental study and comparison of a heuristic logistic, Markov and quantum-like model. Cogn. Psychol. 117:101262. doi: 10.1016/j.cogpsych.2019.101262, PMID: 31865226

[ref5] Bruza P. D. Wang Z. Busemeyer J. R. (2015). Quantum cognition: a new theoretical approach to psychology. Trends Cogn. Sci. 19, 383–393. doi: 10.1016/j.tics.2015.05.001, PMID: 26058709

[ref6] Busemeyer J. R. Wang Z. Lambert-Mogiliansky A. (2009). Empirical comparison of Markov and quantum models of decision making. J. Math. Psychol. 53, 423–433. doi: 10.1016/j.jmp.2009.03.002

[ref7] Busemeyer J. R. Wang Z. Townsend J. T. Eidels A. (2015). The Oxford Handbook of Computational and Mathematical Psychology. Oxford: Oxford University Press.

[ref8] Curtis C. E. Lee D. (2010). Beyond working memory: the role of persistent activity in decision making. Trends Cogn. Sci. 14, 216–222. doi: 10.1016/j.tics.2010.03.006, PMID: 20381406 PMC2883296

[ref9] da Silva F. L. (2013). EEG and MEG: relevance to neuroscience. Neuron 80, 1112–1128. doi: 10.1016/j.neuron.2013.10.017 24314724

[ref10] Hajcak G. Moser J. S. Holroyd C. B. Simons R. F. (2006). The feedback-related negativity reflects the binary evaluation of good versus bad outcomes. Biol. Psychol. 71, 148–154. doi: 10.1016/j.biopsycho.2005.04.001, PMID: 16005561

[ref11] He Z. Jiang W. (2018a). An evidential Markov decision making model. Inf. Sci. 467, 357–372. doi: 10.1016/j.ins.2018.08.013

[ref12] He Z. Jiang W. (2018b). An evidential dynamical model to predict the interference effect of categorization on decision making results. Knowl.-Based Syst. 150, 139–149. doi: 10.1016/j.knosys.2018.03.014

[ref13] Hewig J. Kretschmer N. Trippe R. H. Hecht H. Coles M. G. Holroyd C. B.. (2011). Why humans deviate from rational choice. Psychophysiology 48, 507–514. doi: 10.1111/j.1469-8986.2010.01081.x, PMID: 20667034

[ref14] Huang Z. Yang L. Jiang W. (2019). Uncertainty measurement with belief entropy on the interference effect in the quantum-like Bayesian networks. Appl. Math. Comput. 347, 417–428. doi: 10.1016/j.amc.2018.11.036

[ref15] Jain S. Mittra R. Wiart J. (2015). Full wave modeling of brain waves as electromagnetic waves. Prog. Electromagn. Res. 151, 95–107. doi: 10.2528/PIER15011404

[ref160] Kahneman D. Tversky A. (1979). Prospect theory: an analysis of decision under risk. Econometrica 47, 263–291. doi: 10.2307/1914185

[ref16] Klir G. J. Ramer A. (1990). Uncertainty in the Dempster-Shafer theory: a critical re-examination. Int. J. Gen. Syst. 18, 155–166. doi: 10.1080/03081079008935135

[ref17] Kraemer P. M. Weilbächer R. A. Fontanesi L. Gluth S. (2020). “Neural bases of financial decision making: from spikes to large-scale brain connectivity,” in Psychological Perspectives on Financial Decision Making. eds. Zaleskiewicz T. Traczyk J. (Cham, Switzerland: Springer), 3–19.

[ref18] Kühberger A. Komunska D. Perner J. (2001). The disjunction effect: does it exist for two-step gambles? Organ. Behav. Hum. Decis. Process. 85, 250–264. doi: 10.1006/obhd.2000.2942, PMID: 11461201

[ref19] Lambdin C. Burdsal C. (2007). The disjunction effect reexamined: relevant methodological issues and the fallacy of unspecified percentage comparisons. Organ. Behav. Hum. Decis. Process. 103, 268–276. doi: 10.1016/j.obhdp.2006.04.001

[ref20] Li S. (2004). Equate-to-differentiate approach: an application in binary choice under uncertainty. Cent. Eur. J. Oper. Res. 12, 269–294.

[ref21] Li S. Taplin J. E. (2002). Examining whether there is a disjunction effect in prisoner’s dilemma games. Chin. J. Psychol. 44, 25–46.

[ref22] Mao W. Wang Y. (2011). The processing of visual double-feature conflict under different attentive conditions: an event related potential study. Chin. Ment. Health J. 25, 150–155. doi: 10.3969/j.issn.1000-6729.2011.02.016

[ref23] McFadden J. (2020). Integrating information in the brain’s EM field: the cemi field theory of consciousness. Neurosci. Conscious. 2020:niaa016. doi: 10.1093/nc/niaa016, PMID: 32995043 PMC7507405

[ref24] Moreira C. Wichert A. (2016). Quantum-like Bayesian networks for modeling decision making. Front. Psychol. 7:11. doi: 10.3389/fpsyg.2016.00011, PMID: 26858669 PMC4726808

[ref25] Moreira C. Wichert A. (2017). Exploring the relations between quantum-Like Bayesian networks and decision-making tasks with regard to face stimuli. J. Math. Psychol. 78, 86–95. doi: 10.1016/j.jmp.2016.10.004

[ref26] Moreira C. Wichert A. (2018). Are quantum-like Bayesian networks more powerful than classical Bayesian networks? J. Math. Psychol. 82, 73–83. doi: 10.1016/j.jmp.2017.11.003

[ref27] Pockett S. (2012). The electromagnetic field theory of consciousness: a testable hypothesis about the characteristics of conscious as opposed to non-conscious fields. J. Conscious. Stud. 19, 191–223.

[ref28] Rashkovskiy S. Khrennikov A. (2020). Psychological ‘double-slit experiment’ in decision making: quantum versus classical. Biosystems 195:104171. doi: 10.1016/j.biosystems.2020.104171, PMID: 32485348

[ref29] Suo T. Feng T. Luo J. Luo Y. Li H. (2012). Positive outcome evaluation is modulated by closeness of outcome: an ERP research. Acta Psychol. Sin. 44, 1047–1057. doi: 10.3724/SP.J.1041.2012.01047

[ref30] Suppes P. Barros J. Oas G. (2012). Phase-oscillator computations as neural models of stimulus-response conditioning and response selection. J. Math. Psychol. 56, 95–117. doi: 10.1016/j.jmp.2012.01.001

[ref31] Surov I. A. Pilkevich S. V. Alodjants A. P. Khmelevsky S. V. (2019). Quantum phase stability in human cognition. Front. Psychol. 10:929. doi: 10.3389/fpsyg.2019.00929, PMID: 31114524 PMC6503077

[ref32] Surov I. A. Semenenko E. Platonov A. V. Bessmertny I. A. Galofaro F. Toffano Z.. (2021). Quantum semantics of text perception. Sci. Rep. 11:4193. doi: 10.1038/s41598-021-83490-9, PMID: 33603018 PMC7893056

[ref33] Torres A. Catena A. Cándido A. Maldonado A. Megías A. Perales J. C. (2013). Cocaine dependent individuals and gamblers present different associative learning anomalies in feedback-driven decision making: a behavioral and ERP study. Front. Psychol. 4:122. doi: 10.3389/fpsyg.2013.00122, PMID: 23516173 PMC3600659

[ref34] Townsend J. T. Silva K. M. Spencer-Smith J. Wenger M. J. (2000). Exploring the relations between categorization and decision making with regard to realistic face stimuli. Pragmat. Cogn. 8, 83–105. doi: 10.1075/pc.8.1.05tow

[ref35] Tversky A. Shafir E. (1992). The disjunction effect in choice under uncertainty. Psychol. Sci. 3, 305–310. doi: 10.1111/j.1467-9280.1992.tb00678.x

[ref36] Van’t Wout M. Kahn R. S. Sanfey A. G. Aleman A. (2006). Affective state and decision-making in the ultimatum game. Exp. Brain Res. 169, 564–568. doi: 10.1007/s00221-006-0346-5 16489438

[ref37] Wang Z. Busemeyer J. R. (2016). Interference effects of categorization on decision making. Cognition 150, 133–149. doi: 10.1016/j.cognition.2016.01.019, PMID: 26896726

[ref38] Wang Y. Huang L. Zhang Z. Song J. Bai L. (2014a). Kindness or hostility? Brain dynamics of understanding interactive intentions of other people. Sci. Sin. Vitae 44, 736–746. doi: 10.1360/052013-20

[ref39] Wang Z. Solloway T. Shiffrin R. M. Busemeyer J. R. (2014b). Context effects produced by question orders reveal quantum nature of human judgments. PNAS 111, 9431–9436. doi: 10.1073/pnas.1407756111, PMID: 24979797 PMC4084470

[ref40] Webb R. Glimcher P. W. Louie K. (2020). The normalization of consumer valuations: context-dependent preferences from neurobiological constraints. Manag. Sci. 67, 93–125. doi: 10.1287/mnsc.2019.3536

[ref41] Wichert A. Moreira C. Bruza P. (2020). Balanced quantum-like bayesian networks. Entropy 22:170. doi: 10.3390/e22020170, PMID: 33285945 PMC7516592

[ref42] Xin X. Li Y. Bi Y. Yan B. (2019). Quantum decision-making model based on equate-to-differentiate method: explanation for the disjunction effect in prisoner’s dilemma. Acta Psychol. Sin. 51, 724–733. doi: 10.3724/SP.J.1041.2019.00724

[ref43] Yeung N. Sanfey A. G. (2004). Independent coding of reward magnitude and valence in the human brain. J. Neurosci. 24, 6258–6264. doi: 10.1523/JNEUROSCI.4537-03.2004, PMID: 15254080 PMC6729539

[ref44] Yukalov V. I. Sornette D. (2011). Decision theory with prospect interference and entanglement. Theory Decis. 70, 283–328. doi: 10.1007/s11238-010-9202-y

